# Epidemiological Investigation and Molecular Characterization of Chicken Infectious Anemia Virus in Broilers in Liaoning Province, China

**DOI:** 10.3390/vetsci12111031

**Published:** 2025-10-24

**Authors:** Yufu Li, Zhenyu Chen, Yiyang Huang, Shuang Hu, Qiufeng Lv, Peng Zhang

**Affiliations:** 1 College of Animal Science and Veterinary Medicine, Shenyang Agricultural University, Shenyang 110866, China; 2 College of Life Engineering, Shenyang Institute of Technology, Fushun 113122, China

**Keywords:** chicken anemia virus, epidemiological investigation, genome, recombination analysis, China, Liaoning Province

## Abstract

Chicken anemia virus (CAV) targets the immune system of chickens, causing anemia and atrophy of the bone marrow and lymphoid organs, resulting in significant damage to poultry farming. From April 2024 to March 2025, a total of 359 liver samples were collected from broiler farms in 11 cities across Liaoning Province. CAV was detected using PCR, and 16 complete genome sequences of CAV isolates from different regions were analyzed through phylogenetic and recombination analyses. The overall CAV positivity rate was 13.9%, with spring identified as the peak season. Phylogenetic analysis and genotyping revealed that ten CAV strains clustered within subgroup C1, while the remaining six were distributed among subgroups C2 and C3, as well as Group D. Specific mutations were observed among the VP1 protein genotypes, including mutations previously associated with increased pathogenicity and transmission. One isolate, LN2511, was identified as a potential recombinant strain with its parents CAV-Shanxi7 and CAV-EG-13. During 2024–2025, CAV infection remained prevalent in Liaoning Province, with subtype C1 being the dominant genotype. Amino acid sequence analysis indicated the presence of highly pathogenic strains across the province. These findings fill a knowledge gap regarding CAV infection status and evolutionary trends in chicken populations in Liaoning, China.

## Simple Summary

Chicken anemia virus (CAV) is a poultry virus that attacks chickens’ immune systems, leading to severe anemia and damage to immune organs. This causes major economic losses for chicken farms. To understand the current situation and types of CAV in Liaoning Province, China, we collected 359 liver samples from broiler chickens across 11 cities between April 2024 and March 2025. Using genetic testing methods, we found that 13.9% of the samples were infected, with spring being the high-risk season. By analyzing 16 complete virus genomes, we discovered that the main circulating strains belonged to genetic subgroup C1, while other subtypes were also present. Some virus strains carried specific mutations linked to higher disease severity and spread. We also identified one new recombinant virus strain. This study provides important information on the status and genetic evolution of CAV in Liaoning, which will help in monitoring the virus and developing better prevention strategies to protect poultry health and support local farmers.

## 1. Introduction

Chicken infectious anemia (CIA) is an immunosuppressive disease in poultry caused by chicken anemia virus (CAV). It primarily affects chicks under 2 to 3 weeks of age that lack maternal antibodies [1]. Clinically, CIA is characterized by aplastic anemia, growth retardation, and atrophy of the bone marrow and lymphoid organs [2]. Since CAV primarily targets the chicken’s immune system, the disease manifests as aplastic anemia, growth retardation, lymphoid tissue atrophy, and immunosuppression. This immunosuppression increases the bird’s susceptibility to secondary infections caused by viruses, bacteria, or fungi, thereby making co-infections with other pathogens more likely [3].

CAV belongs to the family Circoviridae and the genus Gyrovirus. It is a non-enveloped, circular, single-stranded DNA virus [4]. Its genome consists of a single-stranded circular DNA of approximately 2.3 kb and contains three major overlapping open reading frames (ORFs) [5]. ORF1 encodes the only capsid protein, viral protein 1 (VP1), which is associated with viral virulence and replication [6]. ORF2 encodes viral protein 2 (VP2), a scaffold protein that also acts as a helper protein for VP1, assisting in its proper folding during virion assembly [7]. ORF3 encodes the viral protein 3 (VP3), a non-structural protein that serves as an apoptosis inducer. It is believed to play a significant role in the apoptosis of thymic lymphocytes and primitive hematopoietic cells [5].

Since CAV was first reported in Japan in 1976, it has spread widely among chicken populations around the world [8]. Recent epidemiological investigations indicate that CAV infections remain a serious concern globally, with infection rates ranging from 8.08% to 85% in different countries [9,10,11]. In recent years, CAV has been identified in multiple provinces throughout China, exhibiting a rising incidence and an increasingly severe epidemic situation, which poses a significant threat to the country’s poultry industry [12,13]. Since the 1990s, molecular epidemiological studies of CAV in China have mainly focused on southern provinces, while little has been reported on the molecular evolution of CAV in Liaoning Province in northeastern China. As a major poultry-producing region adjacent to the Korean Peninsula, with broiler exports primarily to South Korea, Russia, and EU countries, northeast China holds strategic importance [14]. This study conducted a molecular epidemiological investigation and characterization of chicken anemia virus (CAV) in chicken flocks across various regions of Liaoning Province from April 2024 to March 2025. The research involved sequencing CAV strains from chicken samples, performing phylogenetic analysis, and conducting recombination analysis. The aim was to enhance our understanding of CAV infection in Liaoning and to provide updated insights into the genetic evolution of the virus.

## 2. Materials and Methods

### 2.1. Sample Collection, Nucleic Acid Extraction, and CAV Detection

From April 2024 to March 2025, a total of 359 liver samples were collected from 105 poultry farms across 11 cities in Liaoning Province. These samples were obtained from broiler chickens aged 3 to 6 weeks that had not been vaccinated against infectious anemia. These farms were located in Anshan, Benxi, Chaoyang, Yingkou, Dalian, Jinzhou, Fushun, Liaoyang, Dandong, Tieling, and Shenyang (Table 1). These samples were initially sent to Shenyang Aiyou Biotechnology Co., Ltd. for the surveillance of other diseases. All samples were stored at −80 °C until analysis. Viral nucleic acids were extracted from all liver samples using the TIANamp Virus DNA/RNA Kit (DP315) provided by Tiangen Biotech Co., Ltd. (TransGen, Beijing, China). The chicken anemia virus vaccine strain (CAV-VAC), purchased from Merck & Co., Inc. (Rahway, NJ, USA), was used as the positive control in PCR detection.

### 2.2. PCR Amplification and Sequencing of the Full-Length Genome

Sixteen CAV-positive samples were selected for full-length genome amplification. The primers used for CAV full-genome amplification were as follows [13]: CAV1F (5′-GCATTCCGAGTGGTTACTATTCC-3′) and CAV1R (5′-CGTCTTGCCATCTTACAGTCTTAT-3′) amplified an 842 bp fragment. CAV2F (5′-CGAGTACAGGGTAAGCGAGCTAAA-3′) and CAV2R (5′-TGCTATTCATGCAGCGGACTT-3′) amplified a 990 bp fragment. CAV3F (5′-GAAAATGAGACCCGACGAGCAACAG-3′) and CAV3R (5′-GATTCGTCCATCTTGACTTTCTGTG-3′) amplified a 737 bp fragment. These three sets of primers were used together to amplify the complete genome of CAV. The PCR reaction was performed in a 50 μL volume containing 25 μL of 2× Taq PCR Star Mix, 1.5 μL of each primer (10 μmol/L), 3 μL of DNA template, and 19 μL of nuclease-free water. The PCR cycling conditions were as follows: initial denaturation at 95 °C for 5 min; 35 cycles of 95 °C for 30 s, 65 °C for 30 s, and 72 °C for 75 s; followed by a final extension at 72 °C for 10 min. PCR product purification and cloning were carried out using the method described by Teng et al. [14]. Cloning vectors containing the target gene were then sent to a sequencing facility (Sangon Biotech, Shanghai, China) for sequencing.

### 2.3. Phylogenetic Tree and Molecular Characteristics of CAV

A total of 62 CAV genome sequences were downloaded from GenBank (https://www.ncbi.nlm.nih.gov) for use as reference strains (Table 2). Among these, 28 sequences were from China and were published between 2005 and 2022. The remaining 34 sequences included 6 from South Korea, published in 2022. The rest originated from the United States, Tunisia, Malaysia, Japan, Italy, Egypt, the United Kingdom, Australia, and Argentina, and were published between 1991 and 2019. The sequences obtained via Sanger sequencing were assembled using SeqMan Pro 11.1.0(59), resulting in 16 complete CAV genome sequences. A phylogenetic tree was constructed based on these 16 full-length sequences using the neighbor-joining method in MEGA 11.0.13, with 1000 bootstrap replications. CAV strains were labeled in the format: Country/GenBank accession number/Strain name/Year for consistency in the phylogenetic tree. The amino acid sequences of the VP1, VP2, and VP3 genes, inferred from both the 16 sequences obtained in this study and the 62 reference sequences, were analyzed and aligned using DNAStar 11.1.0(59). Comparative analyses and alignment reports were generated accordingly.

### 2.4. Recombination Analysis

In this study, the 16 chicken anemia virus (CAV) genome sequences were aligned with 62 reference full-length CAV genomes using MEGA 11. The alignment results were then imported into the Recombination Detection Program 5 (RDP5) software. Using seven independent methods—RDP, GENECONV, MaxChi, BootScan, Chimera, SiScan, and 3Seq [33,34,35,36,37,38,39,40,41]—we assessed potential recombination events [40]. All methods were run with default settings, with the maximum acceptable *p*-value threshold set at 0.05. The parental sequences and potential recombination breakpoints were evaluated and identified. A recombination event was deemed positive if it was supported by six or more of the aforementioned methods and had a *p*-value of less than 0.05. In addition, SimPlot version 3.5.1 was used to perform similarity plotting analysis on the putative recombinant sequences, parental strains, and outgroup CAV sequences [41].

## 3. Results

### 3.1. CAV Detection Results

**Figure 1:**
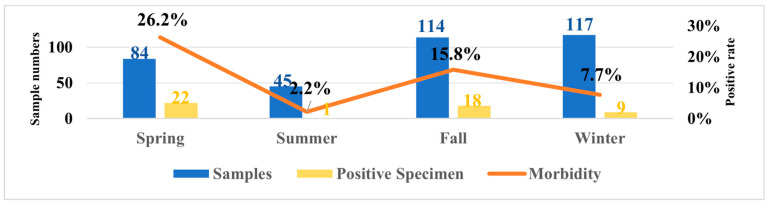
Temporal distribution of samples collected in Liaoning Province from April 2024 to March 2025 and seasonal changes in positivity rates.

The overall CAV positive rate was 13.9% (50/359). As shown in Figure 1, among the 50 CAV-positive samples, the positivity rates by city were as follows: Shenyang 25.5%, Tieling 9.84%, Fuxin 33.3%, Liaoyang 6.7%, Dalian 9.1%, Jinzhou 10%, and Dandong 3.8%. No CAV-positive cases were detected in Benxi, Anshan, Yingkou, and Fushun. Additionally, epidemiological results indicated the CAV prevalence in Liaoning Province by season as: spring 26.2% (22/84), summer 2.3% (1/44), autumn 15.8% (18/114), and winter 7.7% (9/117).

### 3.2. CAV Phylogenetic Tree Analysis

**Figure 2:**
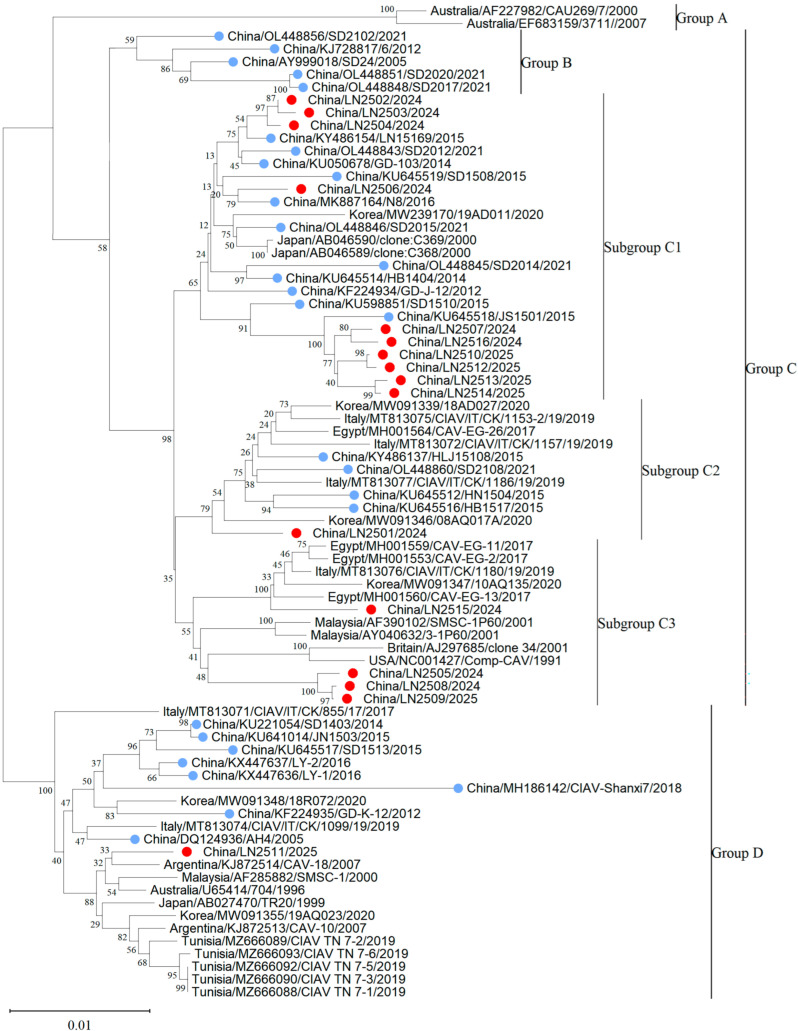
Phylogenetic tree of 16 full-length CAV genome sequences obtained in this study and 62 full-length CAV reference sequences downloaded from GenBank. Red circles indicate sequences obtained from this study. Blue circles for other Chinese strains.

**Figure 3:**
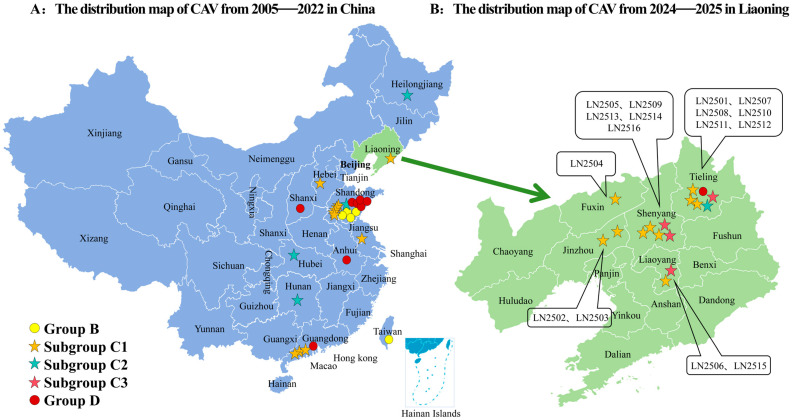
Distribution map of CAV strains based on available data in this study: (**A**) Distribution of CAV strains across different geographic regions in China from 2005 to 2022. (**B**) Distribution of CAV strains in Liaoning Province from 2024 to 2025 based on available sequences. CAV genotype distributions by province are marked with (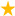 subgroups C1, 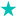 subgroups C2 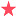 subgroups C3) and (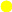 groups B, 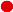 groups D).

In this study, 16 CAV genome sequences, each 2298 bp in length, were obtained by sequencing and analyzed together with 62 reference CAV strains for phylogenetic analysis. Based on the adjacency method in MEGA 11, the phylogenetic tree obtained from 1000 bootstrap replicates divides CAV into four major branches and three minor branches according to genetic distance. CAV strains were grouped into four major clades: Group A (*n* = 2), Group B (*n* = 5), Group C (*n* = 48), and Group D (*n* = 23) (Figure 2). Furthermore, Group C could be subdivided into three distinct subgroups: C1, C2, and C3. Based on the Bootstrap method, 1000 calculations were conducted to obtain the genetic distances among groups A, B, C, and D (Supplementary Table S1). It can be observed that the genetic distance between Group A and Groups B, C, and D is relatively far, while the genetic distances among the three subgroups C1, C2, and C3 are relatively close. The phylogenetic analysis indicated that CAV genotypes in China show clear geographic characteristics, whereas CAV strains within Liaoning Province were mainly clustered in the C1 subgroup. The phylogenetic tree also revealed that among 44 Chinese CAV sequences from 2005 to 2021, 11.4% (5/44) formed a unique branch (Group B) distinct from CAV sequences from other countries. Additionally, of 44 sequences from 2014 to 2025, 47.7% (21/44) clustered in the C1 subgroup. From 2005 to 2020, the C1 subgroup (*n* = 24) was the predominant genotype in China, with 87% (21/24) of strains originating from China. All 16 complete CAV genome sequences obtained in this study clustered within Group C but showed distinct subgroup classifications: 62.5% (10/16) belonged to the C1 subgroup, 25% (4/16) to the C3 subgroup, 6.3% (1/16) to the C2 subgroup, and 6.3% (1/16) to Group D. Among the 44 Chinese CAV sequences analyzed, most were located in Group B and the C1 subgroup; however, Chinese sequences were present in all groups except Group A. CAV strains from the United Kingdom, United States, Italy, and Egypt mainly clustered in the C3 subgroup, while Korean CAV strains primarily belonged to the C2 subgroup and Group D. Group D included 9 Chinese sequences and 14 sequences from Korea, Italy, Argentina, Malaysia, Japan, and Tunisia. Group B consisted of 5 Chinese sequences, and Group A included 2 sequences from Australia (Figure 3).

### 3.3. Molecular Characteristic Analysis

The nucleotide sequence homology among the 16 CAV genome sequences obtained in this study ranged from 96.6% to 99.9%. When compared with 62 reference CAV genome sequences from GenBank, the nucleotide sequence identity of the complete genomes ranged from 94.2% to 99.6%. The isolate LN2502 showed the highest similarity (99.6%) to the 2017 Liaoning isolate LN15169, while isolate LN2506 showed the lowest similarity (94.2%) to the 2018 Shanxi isolate CAV-Shanxi7.

Based on the genotyping from phylogenetic analysis, the amino acid sequences of VP1, VP2, and VP3 from all strains in groups A, B, C (including subgroups C1, C2, and C3), and D were compared. The 16 CAV VP1 protein sequences obtained in this study showed amino acid homology ranging from 97.1% to 100%, with isolates LN2508 and LN2509 having identical VP1 sequences. The amino acid sequence identity among the 16 CAV VP1 sequences and the 62 VP1 sequences retrieved from GenBank ranged from 95.8% to 100%. Comparisons of the VP2 and VP3 sequences derived from the 16 CAV strains in this study with the 62 reference strains revealed amino acid sequence similarities ranging from 95.6% to 100% for VP2 and from 94.4% to 100% for VP3.

**Figure 4:**
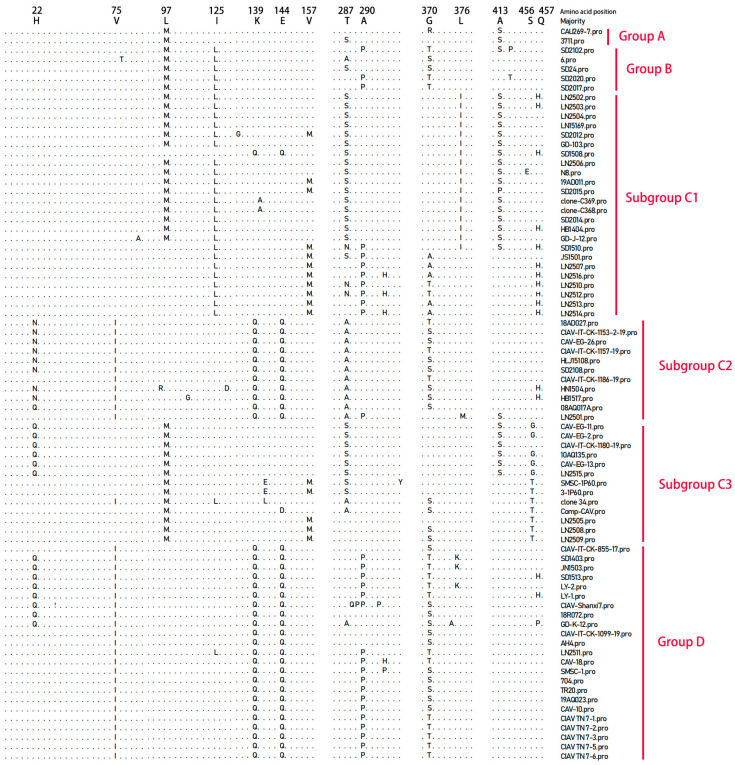
Analysis of major amino acid variation sites in the VP1 gene of chicken anemia virus.

By comparing the VP1 amino acid sequences of the 16 CAV strains examined in this study with those of 62 reference CAV strains, we identified the primary amino acid variations in VP1, as illustrated in the alignment results shown in Figure 4. The mutation sites varied among the different genotypes. Notably, the amino acid residue at position 370 in VP1 displayed a relatively high mutation frequency, occurring in approximately 61.5% (48 out of 78) of the total VP1 amino acid sequences. This mutation at position 370 predominantly occurred in Group B, subgroup C2, and Group D. Among the 78 VP1 amino acid sequences analyzed, 30 contained glycine (Gly) at position 370, while the remaining 48 sequences exhibited alanine (Ala), threonine (Thr), serine (Ser), or arginine (Arg) at this position.

Amino acid variations in Group A were predominantly observed at positions 97 (Leucine [Leu] → Methionine [Met]) and 413 (Alanine [Ala] → Serine [Ser]), both exhibiting mutation frequencies of 100% at these sites. In Group B, mutation sites were primarily at positions 97 (Leu → Met), 125 (Isoleucine [Ile] → Leucine [Leu]), and 370 (Glycine [Gly] → Threonine [Thr] or Serine [Ser]), all with mutation frequencies of 100%. In subgroup C1, amino acid variations were mainly at positions 125 (Ile → Leu), 287 (Threonine [Thr] → Serine [Ser] or Asparagine [Asn]), 376 (Leucine [Leu] → Isoleucine [Ile]), and 413 (Alanine [Ala] → Serine [Ser] or Proline [Pro]), with mutation frequencies of 95.8%, 83.3%, 70.8%, and 70.8%, respectively. In subgroup C2, amino acid substitutions were mainly at positions 139 (Lysine [Lys] → Glutamine [Gln]), 144 (Glutamic acid [Glu] → Glutamine [Gln]), 287 (Threonine [Thr] → Alanine [Ala]), and 370 (Glycine [Gly] → Threonine [Thr] or Serine [Ser]), each with a mutation frequency of 100%. In subgroup C3, amino acid substitutions were mainly concentrated at positions 97 (Leucine [Leu] → Methionine [Met]), 287 (Threonine [Thr] → Serine [Ser] or Alanine [Ala]), and 456 (Serine [Ser] → Glycine [Gly] or Threonine [Thr]), with mutation frequencies of 100%, 76.9%, and 92.3%, respectively. In Group D, amino acid substitutions were mainly found at positions 75 (Valine [Val] → Isoleucine [Ile]), 139 (Lysine [Lys] → Glutamine [Gln]), 144 (Glutamic acid [Glu] → Glutamine [Gln]), 290 (Alanine [Ala] → Proline [Pro]), and 370 (Glycine [Gly] → Threonine [Thr] or Serine [Ser]), all with mutation frequencies of 100%. Subgroups C2 and D share identical amino acid residues at positions 75, 139, and 144, but differ at positions 287 and 290. These data reflect the uniqueness of the VP1 amino acid sequences at mutation sites in the 16 sequenced strains compared with the 62 reference strains, although further sequence comparisons are needed. Analysis of the VP2 and VP3 amino acid sequences indicates no notable differences among the strains.

In this study, we analyzed amino acid residues at positions 75, 89, 125, 141, 144, and 394 in the VP1 protein related to CAV virulence by comparing highly virulent strains (Cux-1 and C368) with a low virulence strain (C369). For 10 of the CAV VP1 proteins examined, the amino acids at these positions were found to be Val, Thr, Leu, Gln, Glu, and Gln, respectively, which aligns with the residues observed at the same positions in the highly virulent strain C368.As shown in Table 3, in contrast, for the other CAV strains included in this study, the amino acid at position 394 in VP1 was Gln, corresponding to the characteristic residue of the highly virulent strain Cux-1.

### 3.4. Recombination Analysis Results

**Figure 5:**
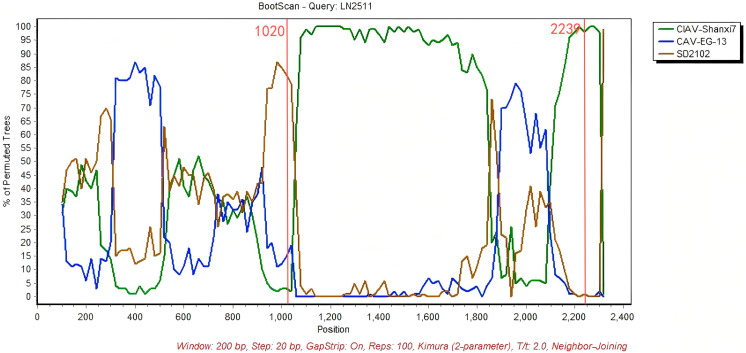
LN2511 was used as the query sequence, with CIAV-Shanxi7 and CAV-EG-13 as the comparison sequences. SD2102 was included as the outgroup. The y-axis represents the percentage of homology calculated using a 200 bp sliding window with a 20 bp step size.The two red lines represent the recombination breakpoints at 1020 and 2239 sites.

With RDP5 software and SimPlot 3.5.1, 16 newly obtained CAV genome sequences and 62 reference complete CAV genomes were analyzed to detect and confirm potential recombination events. If 6 out of the 7 methods for recombination analysis in RDP5 (RDP, GENECONV, MaxChi, BootScan, Chimera, SiScan, and 3Seq) yield positive results (Supplementary Table S1), the recombination event is considered positive. One potential recombinant sequence was identified (isolate LN2511). The results showed that isolating LN2511 from Group D is a potential recombinant strain, supported by six or more algorithms in RDP5. Its major parent was CAV-Shanxi7, and its minor parent was CAV-EG-13. Similarity plot analysis with SimPlot 3.5.1 confirmed that the recombination breakpoints were located at positions 1020 and 2239 (Figure 5).

## 4. Discussion

Chicken infectious anemia virus (CAV) was first reported in Japan in 1979. Following its initial detection in China in 1996, the virus has since spread widely among domestic chicken flocks [42,43]. This study primarily investigated the prevalence of CAV infections across various regions in Liaoning Province. The results revealed significant variation in CAV positivity rates among cities, with Shenyang showing the highest rate at 25.5%. Epidemiological investigations indicate that the infection rate of CAV in broilers in Liaoning Province is significantly higher in spring than in the other three seasons. This seasonal variation may be attributed to the common practice of large-scale broiler production in China, wherein a substantial number of chicks are hatched in early spring [44]. Furthermore, given that CAV can be transmitted both horizontally and vertically, these factors collectively contribute to the observed seasonal disparity. Out of 359 samples, 50 were identified as CAV-positive, and 16 CAV strains were successfully isolated. According to previous studies, Zhang et al. [20] classified CAV into five groups based on 54 partial gene sequences, while Li et al. [18] divided CAV into eight groups using 121 full-length gene sequences. Additionally, Eltahir et al. [19] grouped CAV into four clusters based on 55 complete VP1, VP2, and VP3 gene sequences. Differences in grouping may arise due to variations in analytical methods, reference strains, and the genetic diversity of CAV isolates. In this study, 10 out of the 16 CAV strains isolated from Liaoning Province, China, were found to belong to the C1 subgroup, indicating that C1 is the predominant genotype in this region. The results reveal that among 44 Chinese CAV sequences collected from 2005 to 2025, 47.7% (21/44) were classified into the C1 subgroup, with 87.5% (21/24) of the strains in C1 originating from China. The LN2511 strain isolated in this study belongs to Group D and is genetically similar to CAV strains from Korea, Japan, Australia, and Italy, whereas the other 15 isolates clustered within Group C. The phylogenetic tree further indicates that CAV strains from Liaoning Province are predominantly distributed in the C1 subgroup (10/16), with some presence in subgroups C2, C3, and Group D. This demonstrates a diverse genotypic distribution of CAV in Liaoning Province, underscoring the necessity for region-specific prevention and control strategies tailored to local genotype characteristics. Currently, broiler farms in China immunize breeding stock with the CAV-VAC vaccine. However, due to non-standardized vaccination practices and low vaccine coverage, newly hatched chicks often receive incomplete immunity. Additionally, vaccine contamination contributes to the high prevalence of CAV antibody positivity among Chinese broilers. Therefore, establishing prevention and control standards for CAV in broiler chickens, along with formulating and implementing standardized vaccination protocols, is of paramount importance.

Amino acid residues in the VP1 protein have been demonstrated to be closely associated with the pathogenicity and replication of CAV. Previous studies have confirmed that the amino acid at position 394 of VP1 is critical for CAV virulence: all highly pathogenic clone strains possess glutamine (Gln) at this site, whereas all low-pathogenic clones carry histidine (His) [44]. It has also been reported that mutations at positions 75, 89, 125, 141, and 144 in VP1 lead to attenuation of the Cux-1 strain’s virulence, while the residue at position 394 remains Gln; however, alteration of only one or four of these sites does not reduce the high virulence of Cux-1 [23]. Yamaguchi et al. [44] identified the C368 strain as highly pathogenic. In this study, 10 out of 16 isolated strains have amino acid residues at positions 75, 89, 125, 141, 144, and 394 in VP1 identical to those of C368, suggesting that these 10 strains may represent highly pathogenic CAV isolates. Previous studies have indicated that the amino acid residues at positions 139 and 144 of the VP1 protein influence viral replication and infectivity in cells. CAV strains possessing glutamine (Q) at both these positions exhibit high transmissibility [6,27]. In this study, two isolates (LN2501 and LN2511) were found to have glutamine residues at positions 139 and 144 in their VP1 proteins, which may suggest that these two strains have enhanced transmissibility.

Although CAV is classified as a DNA virus, recombination events occur frequently. These recombination events not only take place between different strains within the same genotype but can also occur across different genotypes. Previous studies have demonstrated that CAV recombination can occur in both coding and non-coding regions, as well as across the boundary between these regions. He et al. [21] confirmed that the SD24 strain is a recombinant and suspected it to be a chimera derived from two different genotypes. Phylogenetic analysis classified SD24, SD2102, SD2020, SD2017, and six other strains into Group B, suggesting that Group B may represent a branch of recombinant strains. However, to validate this hypothesis, additional recombinant strains derived from Groups C and D are required. In this study, in addition to SD24, six recombination events among 62 reference CAV sequences (as listed in Table 2) were utilized to construct the phylogenetic tree. Previous reports have confirmed that these sequences are recombinants originating from various strains within the same genotype [17,20,32]. The recombination event identified in this study involved the CAV-Shanxi7 strain as the primary parent and the CAV-EG-13 strain as the secondary parent. Both strains belong to Group D and subgroup C3, respectively. The recombination breakpoints were identified at nucleotide positions 1020 and 2239. The total length of the three CAV genomes analyzed in this study is 2298 bp, with the VP1 coding region extending from nucleotide 832 to 2181. Consequently, the recombinant region encompasses a portion of the VP1 coding sequence as well as non-coding regions. Notably, this recombinant VP1 segment contains key amino acid sites—positions 75, 89, 125, 141, 144, and 394—that have been reported to influence viral virulence and infectivity [6,25]. Consequently, this recombination event may significantly affect the pathogenicity and transmissibility of CAV.

## 5. Conclusions

In summary, this study is the first to investigate the epidemiology of CAV in various regions of Liaoning Province, China, and to perform full-length genome characterization on 16 selected CAV strains. The overall CAV prevalence in this study was 13.9%. These viruses are categorized into four primary groups: A, B, C (which includes subgroups C1–C3), and D, with subgroup C1 identified as the predominant genotype in Liaoning Province. Analysis of the VP1 protein from the 16 strains revealed variations related to virulence and infectivity. Additionally, a recombination event was detected among the 16 full-length CAV genomes. This study provides molecular epidemiological data and molecular characteristics of CAV strains from different regions of Liaoning Province, contributing to the understanding of CAV genetic evolution and aiding in its prevention and control.

## Figures and Tables

**Table 1:** Information on samples collected from various cities in Liaoning Province and the 16 CAV sequences obtained.

Location	Positive Rate of CAV (Positive Samples/Total Samples)	Accession No.	Strain Name	Year of Collection
Shenyang	25.5% (27/106)	PV815591	LN2509	2025
		PV815585	LN2513	2025
		PV815586	LN2514	2025
		PV815587	LN2516	2024
		PV815588	LN2505	2024
Tieling	9.84% (12/122)	PV815582	LN2510	2025
		PV815583	LN2511	2025
		PV815584	LN2512	2025
		PV815592	LN2501	2024
		PV815581	LN2507	2024
		PV815590	LN2508	2024
Liaoyang	6.7% (2/30)	PV815594	LN2515	2024
		PV815589	LN2506	2024
Jinzhou	10% (2/20)	PV815579	LN2502	2024
		PV815580	LN2503	2024
Fuxin	33.3% (1/3)	PV815593	LN2504	2024
Anshan	23.1% (3/13)	NA	NA	NA
Dalian	9.1% (2/22)			
Dandong	3.8% (1/26)			
Benxi	0% (0/2)			
Yingkou	0% (0/3)			
Fushun	0% (0/12)			

NA: no whole-gene sequencing sample.

**Table 2:** Reference sequences retrieved from GenBank.

Strain	Country/Province	GenBank Accession No.	Reference	Year
SD2102	China/Shandong	OL448856	[15]	2022
SD2020	China/Shandong	OL448851	[15]	2022
SD2015	China/Shandong	OL448846	[15]	2022
SD2014	China/Shandong	OL448845	[15]	2022
SD2012	China/Shandong	OL448843	[15]	2022
SD2108	China/Shandong	OL448860	[15]	2022
SD2017	China/Shandong	OL448848	[15]	2022
19AQ023	Korea	MW091355	[16]	2023
19AD011	Korea	MW239170	[16]	2023
18R072	Korea	MW091348	[16]	2023
18AD027	Korea	MW091339	[16]	2023
10AQ135	Korea	MW091347	[16]	2023
08AQ017A	Korea	MW091346	[16]	2023
CAV-Shanxi7	China/Shanxi	MH186142	NA	2018
N8	China/Guangdong	MK887164	NA	2016
LY-2	China/Shandong	KX447637	NA	2016
LY-1	China/Shandong	KX447636	NA	2016
HLJ15108	China/Heilongjiang	KY486137	[17]	2015
HB1517 ”	China/Hubei	KU645516	[17]	2015
HN1504 ”	China/Hunan	KU645512	[17]	2015
JS1501	China/Jiangsu	KU645518	[18]	2015
LN15169	China/Liaoning	KY486154	[17]	2015
JN1503	China/Shandong	KU641014	[18]	2015
SD1510	China/Shandong	KU598851	[18]	2015
SD1508 ”	China/Shandong	KU645519	[17]	2015
SD1513	China/Shandong	KU645517	NA	2015
GD-103	China/Guangdong	KU050678	NA	2014
HB1404	China/Hubei	KU645514	[19]	2014
SD1403	China/Shandong	KU221054	NA	2014
GD-J-12 ”	China/Guangdong	KF224934	[20]	2012
GD-K-12	China/Hainan	KF224935	[20]	2012
6	China/Taiwan	KJ728817	NA	2012
AH4	China/Anhui	DQ124936	NA	2005
SD24 ”	China/Shandong	AY999018	[21]	2005
clone:C369	Japan	AB046590	[22]	2000
clone:C368	Japan	AB046589	[23]	2000
TR20	Japan	AB027470	NA	1999
CAV-10	Argentina	KJ872513	[24]	2007
CAV-18	Argentina	KJ872514	[24]	2007
CAU269/7	Australia	AF227982	[25]	2000
3711	Australia	EF683159	NA	2007
704	Australia	U65414	NA	1996
clone 34	Britain	AJ297685	[26]	2001
CAV/IT/CK/855/17	Italy	MT813071	[27]	2017
CAV/IT/CK/1153-2/19	Italy	MT813075	[27]	2019
CAV/IT/CK/1157/19	Italy	MT813072	[27]	2019
CAV/IT/CK/1099/19	Italy	MT813074	[27]	2019
CAV/IT/CK/1180/19	Italy	MT813076	[27]	2019
CAV/IT/CK/1186/19	Italy	MT813077	[27]	2019
SMSC-1P60	Malaysia	AF390102	[28]	2001
SMSC-1	Malaysia	AF285882	[29]	2000
3-1P60	Malaysia	AY040632	[28]	2001
CAV TN 7-1	Tunisia	MZ666088	[30]	2019
CAV TN 7-2	Tunisia	MZ666089	[30]	2019
CAV TN 7-3	Tunisia	MZ666090	[30]	2019
CAV TN 7-5	Tunisia	MZ666092	[30]	2019
CAV TN 7-6	Tunisia	MZ666093	[30]	2019
Comp-CAV	USA	NC001427	[31]	1991
CAV-EG-26	Egypt	MH001564	[32]	2017
CAV-EG-13 ”	Egypt	MH001560	[32]	2017
CAV-EG-11	Egypt	MH001559	[32]	2017
CAV-EG-2	Egypt	MH001553	[32]	2017

NA: no reference available. ”: confirmed recombinant sequences.

**Table 3:** Amino acid variations at key VP1 sites among the 16 sequenced strains compared with Cux-1, C368, and C369.

Strain	Amino Acid Position in VP1					
	75	89	125	141	144	394
Cux-1	V	T	I	Q	D	Q
C368	V	T	L	Q	E	Q
C369	V	T	L	Q	E	H
LN2501	I	T	I	Q	Q	Q
LN2502	V	T	L	Q	E	Q
LN2503	V	T	L	Q	E	Q
LN2504	V	T	L	Q	E	Q
LN2505	V	T	I	Q	E	Q
LN2506	V	T	L	Q	E	Q
LN2507	V	T	L	Q	E	Q
LN2508	V	T	I	Q	E	Q
LN2509	V	T	I	Q	E	Q
LN2510	V	T	L	Q	E	Q
LN2511	I	T	L	Q	Q	Q
LN2512	V	T	L	Q	E	Q
LN2513	V	T	L	Q	E	Q
LN2514	V	T	L	Q	E	Q
LN2515	V	T	I	Q	E	Q
LN2516	V	T	L	Q	E	Q

Note: Cux-1, C368, and C369 are reference strains.

## Data Availability

The original contributions presented in the study are included in the article/supplementary material; further inquiries can be directed to the corresponding author.

## Supplementary Materials

The following supporting information can be downloaded at: https://www.mdpi.com/article/10.3390/vetsci12111031/s1, Table S1: Genetic distance among groups and subgroups of CAV phylogenetic tree.

## Abbreviations

The following abbreviations are used in this manuscript:

CAV	Chicken anemia virus
CIA	Chicken infectious anemia
VP1	Viral protein 1
VP2	Viral protein 2
VP3	Viral protein 3
ORFs	Open reading frames
RDP5	Recombination Detection Program 5

## References

[1] Adair B.M. (2000). Immunopathogenesis of chicken anemia virus infection. Dev. Comp. Immunol..

[2] EngstrÃm B.E., Luthman M. (1984). Blue wing disease of chickens: Signs, pathology and natural transmission. Avian Pathol..

[3] McNulty M.S., McIlroy S.G., Bruce D.W., Todd D. (1991). Economic effects of subclinical chicken anemia agent infection in broiler chickens. Avian Dis..

[4] Rosario K., Breitbart M., Harrach B., SegalÃs J., Delwart E., Biagini P. (2017). Revisiting the taxonomy of the family *Circoviridae*: Establishment of the genus *Cyclovirus* and removal of the genus *Gyrovirus*. Arch. Virol..

[5] Rosenberger J.K., Cloud S.S. (1998). Chicken anemia virus. Poult. Sci..

[6] Renshaw R.W., Soiné C., Weinkle T., O’Connell P.H., Ohashi K., Watson S. (1996). A hypervariable region in VP1 of chicken infectious anemia virus mediates rate of spread and cell tropism in tissue culture. J. Virol..

[7] Moeini H., Omar A.R., Rahim R.A., Yusoff K. (2011). Development of a DNA vaccine against chicken anemia virus by using a bicistronic vector expressing VP1 and VP2 proteins of CAV. Comp. Immunol. Microbiol. Infect. Dis..

[8] Monu K., Mousumi B., Manu M., Ashok K. (2025). An updated review on chicken infectious anaemia. World’s Poult. Sci. J..

[9] Tan C., Wang Z., Lei X., Lu J., Yan Z., Qin J. (2020). Epidemiology, molecular characterization, and recombination analysis of chicken anemia virus in Guangdong province, China. Arch. Virol..

[10] Li Y., Yan N., Wang Y., Liu A., Liu C., Lan X. (2021). Molecular evolution and pathogenicity of chicken anemia virus isolates in China. Arch. Virol..

[11] Zhang M., Deng X., Xie Z., Zhang Y., Xie Z., Xie L. (2022). Molecular characterization of chicken anemia virus in Guangxi Province, southern China, from 2018 to 2020. J. Vet. Sci..

[12] Wang X.W., Feng J., Jin J.X., Zhu X.J., Sun A.J., Liu H.Y. (2022). Molecular epidemiology and pathogenic characterization of novel chicken infectious Anemia viruses in Henan Province of China. Front. Vet. Sci..

[13] Pozdniakova E., Pozdniakov V., Brench A. (2019). Development trends and risk factors of meat global exports. Ukr. Food J..

[14] Teng L., Xie Z., Xie L., Liu J., Pang Y., Deng X. (2014). Sequencing and phylogenetic analysis of an avian reovirus genome. Virus Genes.

[15] Liu L., Li Y., Yin M., Zhao P., Guo L., Wang Y. (2022). Genomic characterization of chicken anemia virus in broilers in Shandong Province, China, 2020–2021. Front. Vet. Sci..

[16] Song H., Kim H., Kwon Y., Kim H. (2024). Genetic characterization of chicken infectious anaemia viruses isolated in Korea and their pathogenicity in chicks. Front. Cell. Infect. Microbiol..

[17] Yao S., Tuo T., Gao X., Han C., Yan N., Liu A. (2019). Molecular epidemiology of chicken anaemia virus in sick chickens in China from 2014 to 2015. PLoS ONE.

[18] Li Y., Fang L., Cui S., Fu J., Li X., Zhang H. (2017). Genomic characterization of recent chicken anemia virus isolates in China. Front. Microbiol..

[19] Eltahir Y.M., Qian K., Jin W., Wang P., Qin A. (2011). Molecular epidemiology of chicken anemia virus in commercial farms in China. Virol. J..

[20] Zhang X., Liu Y., Wu B., Sun B., Chen F., Ji J. (2013). Phylogenetic and molecular characterization of chicken anemia virus in southern China from 2011 to 2012. Sci. Rep..

[21] He C.Q., Ding N.Z., Fan W., Wu Y.H., Li J.P., Li Y.L. (2007). Identification of chicken anemia virus putative intergenotype recombinants. Virology.

[22] Todd D., Scott A.N., Ball N.W., Borghmans B.J., Adair B.M. (2002). Molecular basis of the attenuation exhibited by molecularly cloned highly passaged chicken anemia virus isolates. J. Virol..

[23] Yamaguchi S., Imada T., Kaji N., Mase M., Tsukamoto K., Tanimura N. (2001). Identification of a genetic determinant of patho genicity in chicken anaemia virus. J. Gen. Virol..

[24] Rimondi A., Pinto S., Olivera V., Dibárbora M., Pérez-Filgueira M., Craig M.I. (2014). Comparative histopathological and immunological study of two field strains of chicken anemia virus. Vet. Res..

[25] Brown K., Browning G., Scott P., Crabb B. (2000). Full-length infectious clone of a pathogenic Australian isolate of chicken anaemia virus. Aust. Vet. J..

[26] Scott A.N.J., McNulty M.S., Todd D. (2001). Characterisation of a chicken anaemia virus variant population that resists neutralisation with a group-specific monoclonal antibody. Arch. Virol..

[27] Quaglia G., Mescolini G., Catelli E., Berto G., Muccioli F., Lupini C. (2021). Genetic heterogeneity among chicken infectious anemia viruses detected in Italian fowl. Animals.

[28] Chowdhury S.M.Z.H., Omar A.R., Aini I., Hair-Bejo M., Jamaluddin A.A., Md-Zain B.M. (2003). Pathogenicity, sequence and phylogenetic analysis of Malaysian Chicken anaemia virus obtained after low and high passages in MSB-1 cells. Arch. Virol..

[29] Chowdhury S.M., Omar A.R., Aini I., Hair-Bejo M., Jamaluddin A.A., Kono Y. (2002). Isolation, identification and characteriza tion of chicken anaemia virus in Malaysia. J. Biochem. Mol. Biol. Biophys..

[30] Di Francesco A., Quaglia G., Salvatore D., Sakhria S., Catelli E., Bessoussa G. (2021). Occurrence of chicken infectious anemia virus in industrial and backyard tunisian broilers: Preliminary results. Animals.

[31] Noteborn M.H., De Boer G.F., Van Roozelaar D.J., Karreman C., Kranenburg O., Vos J.G. (1991). Characterization of cloned chicken anemia virus DNA that contains all elements for the infectious replication cycle. J. Virol..

[32] Erfan A.M., Selim A.A., Naguib M.M. (2018). Characterization of full genome sequences of chicken anemia viruses circulating in Egypt reveals distinct genetic diversity and evidence of recombination. Virus Res..

[33] Martin D., Rybicki E. (2000). RDP: Detection of recombination amongst aligned sequences. Bioinformatics.

[34] Padidam M., Sawyer S., Fauquet C.M. (1999). Possible emergence of new geminiviruses by frequent recombination. Virology.

[35] Martin D.P., Posada D., Crandall K.A., Williamson C. (2005). A modified bootscan algorithm for automated identification of recom binant sequences and recombination breakpoints. AIDS Res. Hum. Retroviruses.

[36] Maynard S.J. (1992). Analyzing the mosaic structure of genes. J. Mol. Evol..

[37] Posada D., Crandall K.A. (2001). Evaluation of methods for detecting recombination from DNA sequences: Computer simulations. Proc. Natl. Acad. Sci. USA.

[38] Gibbs M.J., Armstrong J.S., Gibbs A.J. (2000). Sister-Scanning: A Monte Carlo procedure for assessing signals in recombinant se quences. Bioinformatics.

[39] Lam H.M., Ratmann O., Boni M.F. (2018). Improved algorithmic complexity for the 3SEQ recombination detection algorithm. Mol. Biol. Evol..

[40] Martin D.P., Murrell B., Golden M., Khoosal A., Muhire B. (2015). RDP4: Detection and analysis of recombination patterns in virus genomes. Virus Evol..

[41] Lole K.S., Bollinger R.C., Paranjape R.S., Gadkari D., Kulkarni S.S., Novak N.G. (1999). Full-length human immunodeficiency virus type 1 genomes from subtype C-infected seroconverters in India, with evidence of intersubtype recombination. J. Virol..

[42] Schat K.A., Van Santen V.L. (2003). Chicken infectious anemia. Dis. Poult..

[43] Shah P.T., Bahoussi A.N., Cui X., Shabir S., Wu C., Xing L. (2023). Genetic diversity, distribution, and evolution of chicken anemia virus: A comparative genomic and phylogenetic analysis. Front. Microbiol..

[44] Zheng L.P., Teng M., Li G.X., Zhang W.K., Wang W.D., Liu J.L., Li L.Y., Yao Y.X., Venugopal N., Luo J. (2022). Current epidemiology and co-infections of avian immunosuppressive and neoplastic diseases in chicken flocks in central China. Viruses.

