# Supplementary material for: Epidemiological Investigation and Molecular Characterization of Chicken Infectious Anemia Virus in Broilers in Liaoning Province, China
Source: Vet Sci. 2025 Oct 24;12(11):1031. doi: 10.3390/vetsci12111031 (PMC12656911; doi:10.3390/vetsci12111031)
Supplement: Supplementary file 1 [file vetsci-12-01031-s001.zip › vetsci-3907802-supplementary.pdf]

Supplementary Table S1. Genetic distance among groups and subgroups of CAV phylogenetic tree

	A	B	C1	C2	C3	D
A						
B	0.047					
C1	0.046	0.026				
C2	0.047	0.027	0.022			
C3	0.047	0.029	0.022	0.022		
D	0.047	0.026	0.037	0.033	0.037	
